# Microsecond Backbone Motions Modulate the Oligomerization of the DNAJB6 Chaperone

**DOI:** 10.1002/ange.202116403

**Published:** 2022-03-19

**Authors:** Emma E. Cawood, G. Marius Clore, Theodoros K. Karamanos

**Affiliations:** ^1^ Astbury Centre for Structural Molecular Biology School of Molecular and Cellular Biology University of Leeds Mount Preston Street Leeds LS2 9JT UK; ^2^ Laboratory of Chemical Physics National Institute of Diabetes and Digestive and Kidney Diseases National Institutes of Health Bethesda MD 20892-0520 USA

**Keywords:** Hsp40 Chaperones, Oligomerization, Protein Correlated Motions, Protein Dynamics, Protein Excited States, Relaxation-Based NMR

## Abstract

DNAJB6 is a prime example of an anti‐aggregation chaperone that functions as an oligomer. DNAJB6 oligomers are dynamic and subunit exchange is critical for inhibiting client protein aggregation. The T193A mutation in the C‐terminal domain (CTD) of DNAJB6 reduces both chaperone self‐oligomerization and anti‐aggregation of client proteins, and has recently been linked to Parkinson's disease. Here, we show by NMR, including relaxation‐based methods, that the T193A mutation has minimal effects on the structure of the β‐stranded CTD but increases the population and rate of formation of a partially folded state. The results can be rationalized in terms of β‐strand peptide plane flips that occur on a timescale of ≈100 μs and lead to global changes in the overall pleat/flatness of the CTD, thereby altering its ability to oligomerize. These findings help forge a link between chaperone dynamics, oligomerization and anti‐aggregation activity which may possibly lead to new therapeutic avenues tuned to target specific substrates.

## Graphical abstract

Chaperone self‐oligomerization that competes with substrate assembly is an important aspect of anti‐aggregation chaperones that remains poorly understood. Using the J domain chaperone DNAJB6, how the Parkinson's related T193A mutation affects chaperone oligomerization has been explored. The C‐terminal domain of DNAJB6b exhibits significant microsecond dynamics that are enhanced in the T193A mutant, leading to a monomeric partially‐folded state.

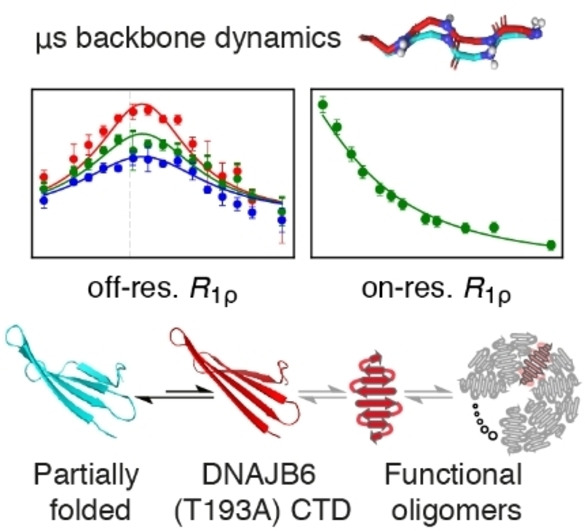

Self‐oligomerization is a key feature of many chaperones that inhibit the aggregation of their client proteins.^[1]^ These chaperones often form co‐aggregates with aggregation‐prone peptides/proteins, leading to increased solubility of their substrates.^[1]^ Since the end‐state homo‐aggregates are often the most stable substrate state, there is an energetic penalty for the formation of chaperone‐substrate complexes. This energetic deficiency is balanced by the fact that that the chaperones alone exist in a high energy state and therefore prefer to interact with their substrates, leading to a gain in free energy upon co‐aggregate formation. These high chemical potential chaperone states are typically manifested as dynamic heterogeneous oligomers.^[2]^ Subunit exchange in these assemblies leads to the capture of aggregated substrates^[3]^ and is the driving force behind co‐aggregate formation. Understanding the molecular basis of chaperone oligomerization/subunit exchange may present an attractive approach for modulating chaperone activity and consequent disease prevention.

The Hsp40 chaperone^[4]^ DNAJB6 is a potent protein aggregation inhibitor, active against amyloid β,^[5]^ Huntingtin‐derived peptides with expanded poly‐glutamine tracts,^[6]^ and α‐synuclein,^[7]^ among others.^[8]^ The short DNAJB6 isoform (DNAJB6b) self‐assembles into polydisperse oligomers ranging from 27 kDa to 1 MDa in size,^[6b]^ which form through interactions mediated by its C‐terminal domain (CTD).^[9]^ We previously showed that isolated CTD constructs are predominantly monomeric at low concentrations but retain key features of full‐length DNAJB6b, including oligomer formation and exchange between monomeric and oligomeric species,^[9b]^ and thus represent ideal constructs to study DNAJB6b self‐assembly and subunit exchange. To describe chemical exchange phenomena involving the isolated CTD we previously used a 4‐state kinetic model in which the major monomeric state (M) dimerizes to form state D, which, in turn, interacts with large CTD oligomers (state DO).^[9b]^ State M can also bind to oligomers, to form state MO, but the population of the MO species is very small and can safely be ignored. Thus, for the purposes of this paper, we consider a simpler 3‐state *M↔D↔DO* model to describe concentration‐dependent chemical exchange in the CTD. At the origin of CTD oligomerization lies a subtle conformational change in the β1 strand, which transitions from a twisted configuration in state M, to a more canonical, “straight” arrangement in the dimer (state D), which is prone to self‐assemble.^[9b]^ We also previously showed that the T193A (or T142A, if using the numbering of ref. [9b]) mutation in strand β1, known to decrease DNAJB6b's ability to inhibit substrate protein aggregation,^[10]^ reduces CTD self‐oligomerization.^[9b]^ Recently, the T193A substitution has also been directly linked to Parkinson's disease^[11]^ suggesting a complex balance between chaperone dynamics, chaperone oligomerization, substrate binding, and disease onset. Here, using solution NMR methods, we study the mechanisms whereby the T193A mutation alters the oligomerization properties of the CTD domain. We show that the threonine to alanine substitution does not affect the CTD structure but has a significant impact on the μs dynamics of residues in the β2 strand that are associated with the formation of a partially folded state. While this state is also detectable in the wild‐type (WT) CTD, it has a reduced population and slower rate of formation. The μs motions are not limited to strand β2, but extend to residues in the hairpins that connect the antiparallel CTD strands, consistent with concerted backbone motions. Overall, our results show that the CTD of DNAJB6b possesses considerable structural plasticity which can modulate its oligomerization state.

The Parkinson's‐related T193A mutation^[11]^ occurs in the middle of the β1 strand, a region that is important for CTD oligomerization.^[9]^ Thus, we first investigated whether the T193A mutation alters the structure of the CTD and affects its ability to self‐associate. When compared to the WT CTD, and excluding the site of mutation, the Cα and Cβ backbone chemical shifts in the T193A CTD are minimally impacted (chemical shift difference, Δδω, <0.2 ppm) (Figure 1A). The Δδω values for backbone N and H_N_ atoms, however, extend further than the T193/A193 site, to residues in the β2 strand (especially those that are hydrogen bonded to strand β1: I203, T205 and R207; Figure 1A), an observation that could suggest weakening of the hydrogen bonds between strands β1 and β2 in the T193A variant.

**Figure 1:**
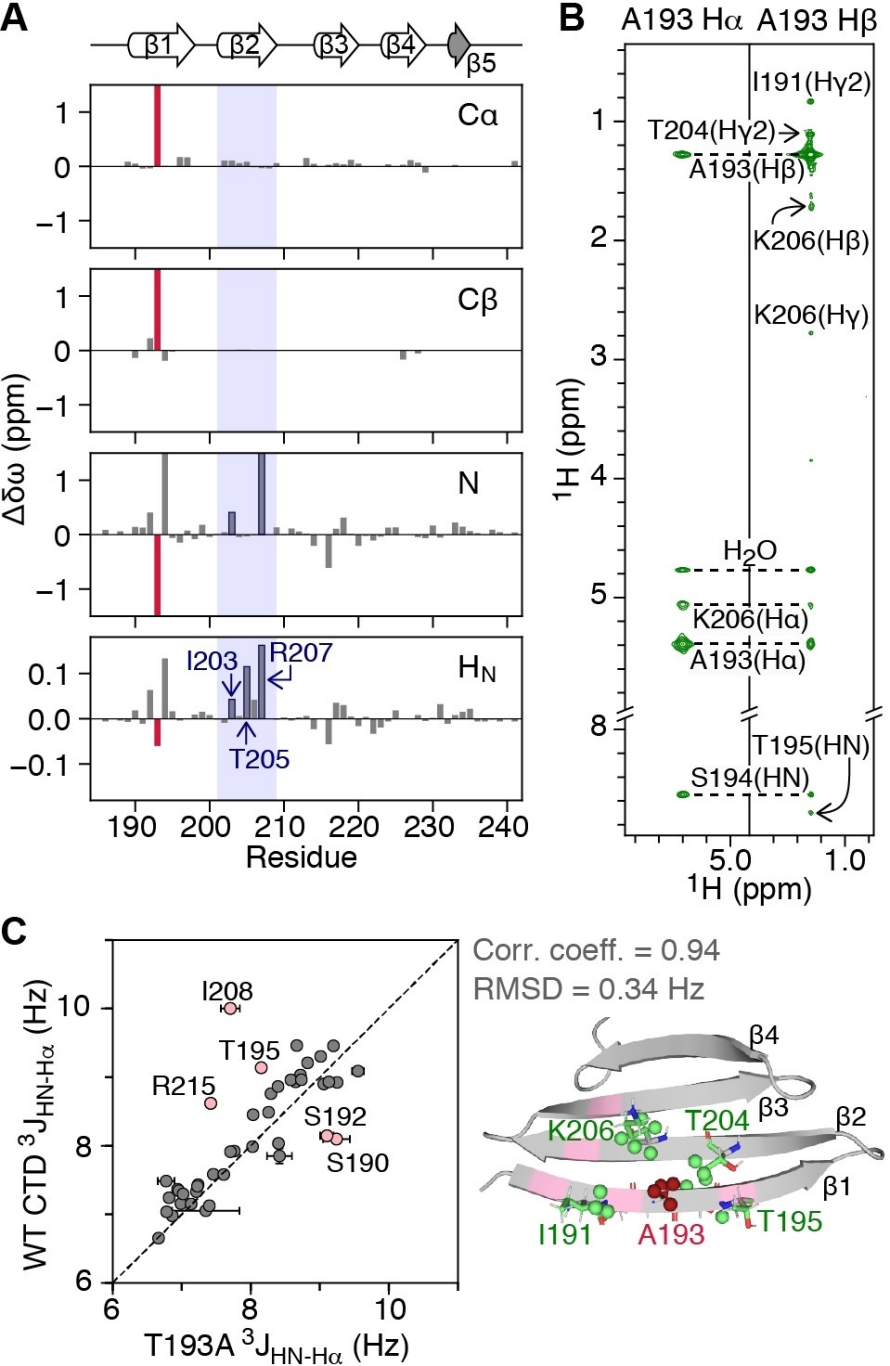
The T193A mutation within the CTD of DNAJB6b does not significantly affect the CTD architecture. A) Chemical shift differences for backbone Cα, Cβ, N, H_N_ atoms of T193A relative to WT CTD (Δδω=δω_WT‐CTD_–δω_T193A_). The T193A site is shown as a red bar. Note that R207 is hydrogen bonded to S192. B) Strips of the 600 MHz non‐uniformly sampled 3D HHC NOE‐HSQC spectrum (mixing time 200 ms) collected on a 500 μΜ U‐[^13^C, ^15^N]‐labeled T193A sample at 298 K. C) Correlation between ^3^ *J*_HN‐Hα_ couplings of T193A and WT CTDs measured using the ARTSY approach^[12]^ (left). Cartoon representation (right) of the refined T193A structure, with residues for which NOEs are observed in (B) shown in green and A193 shown in red. Residues for which coupling constants differ between the two constructs (perhaps due to different amplitude of ms motions^[9b]^) are shown in pink. The correlation coefficient and RMSD exclude the residues in pink that display ms dynamics.

To probe the structure of the T193A CTD in more detail, we used a [^13^C, ^15^N]‐labeled sample to collect a set of 3D HHC NOE‐HSQC and HCC HMQC‐NOE‐HSQC spectra.^[13]^ Long range NOEs connecting A193 with residues in strand β2 were observed as expected for antiparallel β‐strands, and NOEs outside the mutation site are essentially unchanged both in terms of pattern and intensity (Figure 1B), compared to WT CTD.^[9b]^ The correlation coefficient for ^3^ *J*_HN‐Hα_ couplings between the T193A and WT CTDs is 0.80 with a root‐mean‐square deviation (RMSD) of 0.57 Hz (Figure 1C, left). Excluding five residues in the dynamic β1 strand and the β2‐β3 loop that show motions on the ms timescale that differ in amplitude between the two constructs^[9b]^ (pink residues in Figure 1C), increases the correlation coefficient to 0.94 and decreases the RMSD to 0.34 Hz. Finally, refining the structure of the WT CTD to include the T193A mutation using the measured NOEs and ^3^ *J*_HN‐Hα_ couplings results in a structure with a Cα backbone RMSD of 0.9 Å to WT, over the rigid regions of the CTD (Figures 1C, S1A and Table S1).

Although the β‐sheet architecture of the CTD is minimally affected by the T193A substitution, its dynamics are significantly impacted. Figure 2 displays ^15^Ν‐*R*_2_ profiles for WT and T193A CTDs at 288 and 298 K. At 288 K, the ^15^Ν‐*R*_2_ profiles for WT CTD are relatively uniform with the exception of five residues in β‐hairpins (Figure 2, green bars), suggesting that chemical exchange is efficiently supressed by the applied 1.5 kHz spinlock. Significantly higher ^15^Ν‐*R*_2_ values, however, are observed for residues in the β2 strand of T193A, an observation that is independent of protein concentration (Figure 2A). These differences in ^15^Ν‐*R*_2_ between T193A and WT CTDs cannot be explained by an anisotropic diffusion tensor (the value of D_||_/D_⊥_ for the CTD is ≈1.74), nor by lifetime line broadening. Interestingly, raising the temperature from 288 K to 298 K causes residues in the β2 strand of WT CTD to also show elevated ^15^Ν‐*R*_2_ values (Figures 2B and S1B). Together, the data in Figure 2 suggest that there is a fast exchange process between species with different chemical shifts, which involves the β2 strand in both T193A and WT CTDs. However, this process appears to be significantly less prominent and/or slower for the WT CTD since it is only apparent at 298 K.

**Figure 2:**
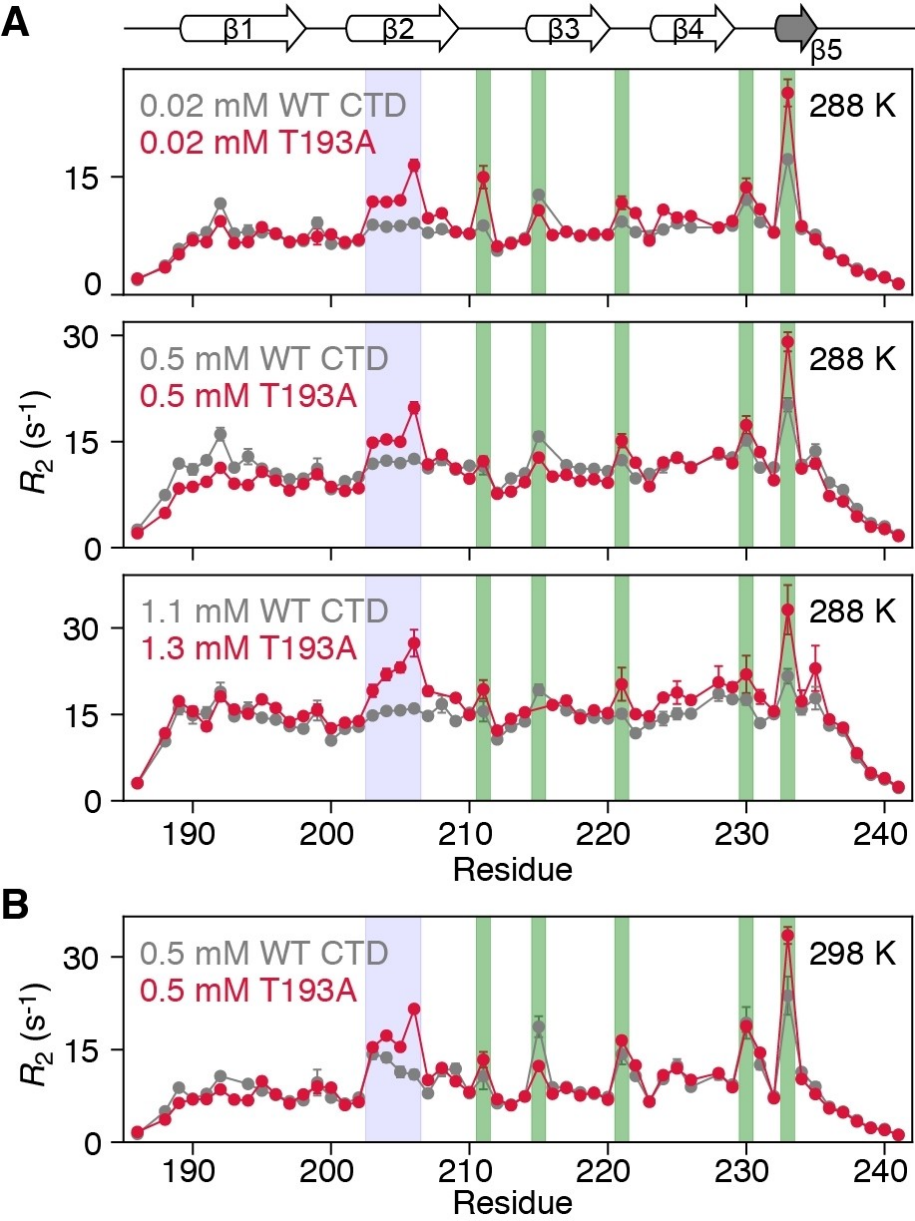
The β2 strand of the T193A CTD shows increased dynamics that are not concentration‐dependent. ^15^N‐*R*_2_ profiles for WT CTD (grey) and T193A (red) at A) 288 K and various concentrations and B) 298 K and 0.5 mM. The data were recorded at 600 MHz with a ^15^N‐*R*_1ρ_ sequence^[14]^ and a 1.5 kHz spinlock field, and then converted to *R*_2_ values. Residues in the β2 strand, hairpins are highlighted with blue, green boxes respectively. If error bars are not visible, they are smaller than the circles representing the experimental data point.

To probe the fast exchange process in more detail, we carried out ^15^N‐ and ^1^H_N_‐*R*_1ρ_ (Figures S2–S4) relaxation dispersion experiments to probe motions on the μs timescale.^[17]^ For the T193A CTD, large on‐resonance ^1^H_N_‐*R*_1ρ_ relaxation dispersion profiles as a function of spinlock RF field strength were observed throughout the protein (Figures 3A, S2A), while off‐resonance ^15^N‐*R*_1ρ_ as a function of offset from the carrier show significant profiles for residues in the β2 strand and hairpins (Figures 3B, S3, Table S2). For the WT CTD, off‐resonance ^15^N‐*R*_1ρ_ profiles are asymmetric due to exchange with large oligomers[^9b^, ^18^] and we therefore made use of on‐resonance experiments shown in Figures 3C and S4A. Initial fits of the relaxation data showed that exchange is fast on the chemical shift timescale, and therefore, only the product of the minor species population (*p*_E_) and the chemical shift difference (Δω_Ε_) between the minor and major species can be determined from the *R*_1ρ_ relaxation dispersion data alone.^[17]^ To decorrelate *p*_E_ and Δω_Ε_, we performed ^15^N‐CPMG (Carr–Purcell–Meiboom–Gill) relaxation dispersion experiments^[19]^ (Figures S2B, S4B, S5, and Supporting Information Materials and Methods).

**Figure 3:**
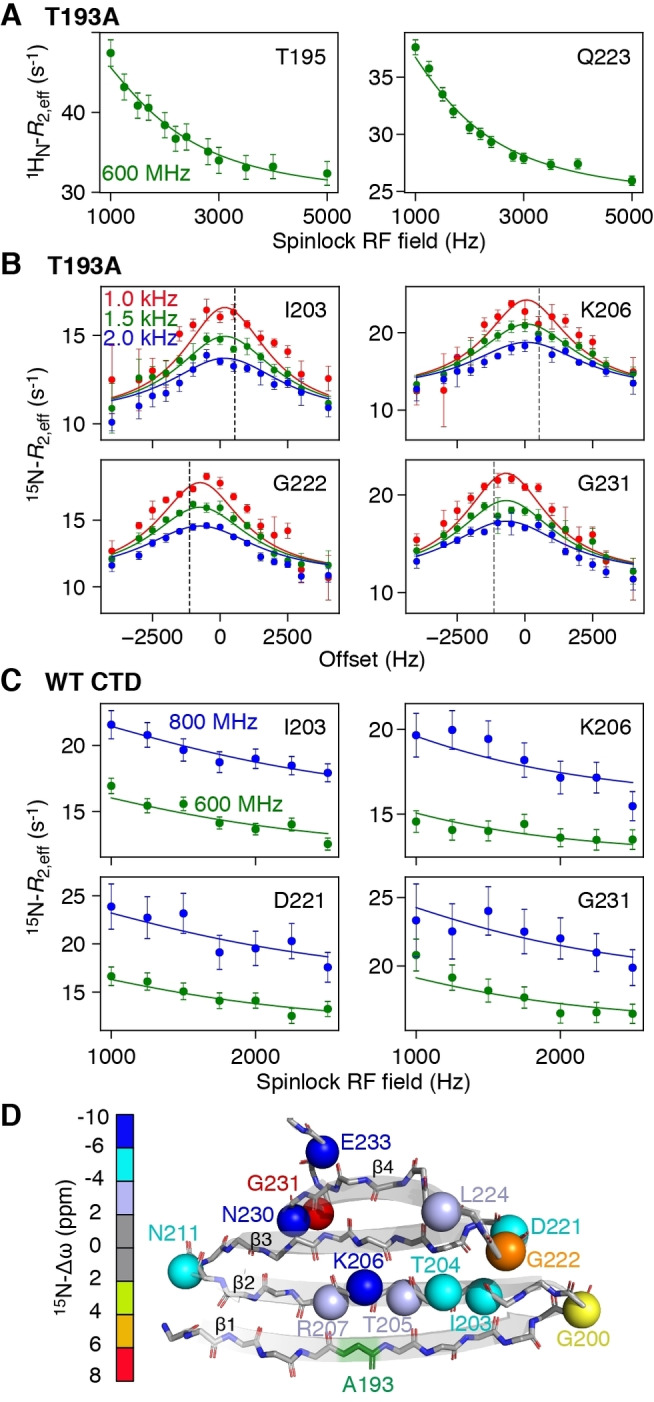
Probing μs dynamics using *R*_1ρ_ relaxation dispersion. A) On‐resonance ^1^H_N_‐*R*_1ρ_ ^[15]^ and B) off‐resonance ^15^N‐*R*_1ρ_ relaxation dispersion^[16]^ profiles for the T193A CTD at 600 MHz. The different colors denote different spin lock radio‐frequency (RF) field strengths and the dashed line corresponds to the offset of the major state resonance from the carrier. C) On‐resonance ^15^N‐*R*_1ρ_ relaxation dispersion profiles for WT CTD collected at 600 (green) and 800 MHz (blue). D) Cartoon representation of the structure of the T193A CTD. The backbone nitrogen atoms of residues that show μs dynamics are depicted as spheres and colored according to their Δω value.

Using the above strategy, and globally fitting the CPMG and *R*_1ρ_ relaxation dispersion data together, we were able to determine Δω values for T193A (Figure 3D) and WT CTD (Tables S2, S3), together with species populations and exchange rates (Figure 4A). We made use of the 4‐state model depicted in Figure 4A, which includes an additional monomeric state (M*) added to the previously defined 3‐state model.^[9b]^ M* interconverts directly with M to give M*↔M↔D↔DO. For WT CTD, the population of M* is ≈0.8 % and the overall exchange rate between M and M* is ≈8000 s^−1^; for the T193A mutant these values are increased to ≈2.5 % and ≈10 000 s^−1^, respectively. The increased population of M* in the T193A mutant effectively decreases the concentration of species D and redirects the CTD equilibria to the off‐pathway M* state, whereas in WT CTD the *M↔D↔DO* oligomerization pathway prevails (Figure 4A). The increased population of the off‐pathway state M*, in combination with the subtle structural changes in state M (Figures 1 and S1A) that lead to a 2‐fold increase in the dimer dissociation constant,^[9b]^ make the T193A mutant less prone to oligomerization.

**Figure 4:**
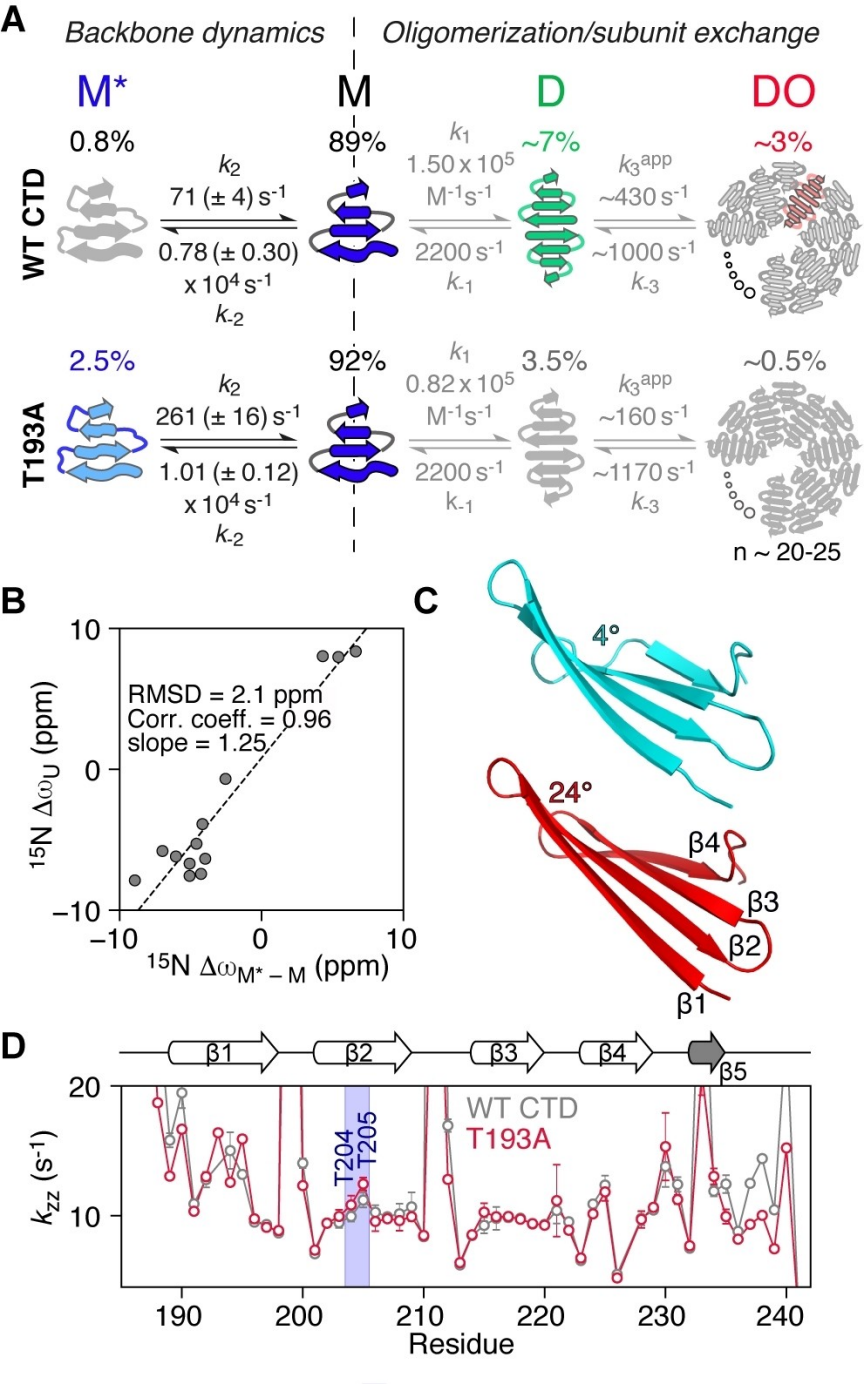
The T193A mutation redirects CTD oligomerization by promoting the formation of a partially folded state. A) 4‐state kinetic model used to fit the relaxation data for WT (top) and T193A (bottom) CTDs. The kinetic parameters for the *M↔D↔DO* oligomerization pathway (grey text) were determined in ref. [9b]. Twisted arrows depict a partially folded structure (M*) or a twisted β1 strand in M.^[9b]^ Straight arrows in D depict a straight β1.^[9b]^ B) Correlation of the fitted chemical shift differences for the M−M* transition (Δω_M‐M*_) with the differences in chemical shift between random coil and folded states (Δω_U_) for the T193A mutant. C) A cartoon representation of strands β1 to β4 in the T193A CTD when the twist angle for residues 203–206 is set to 4° (cyan) or 24° (red) using twistPot (the value observed in the refined T193A structure is ≈20°). A morph between these two structures is provided as Supplementary Video 1. D) *k*_ZZ_ rates obtained using the DÉCOR experiment^[24]^ at 298 K and 600 MHz as a function of residue number for the WT (grey) and T193A (red) CTDs. *k*_zz_, the relaxation rate of H_z_N_z_ two spin order, is dominated by amide proton exchange with solvent (see also Figure S6C).

The fast rate of formation of M* is indicative of backbone reorientation/peptide plane flips on the μs timescale^[20]^ and is consistent with our previous observations that strand β1 exchanges between a “straight” and twisted conformation.^[9b]^ Presumably, hydrogen bonds to strand β2 have to break to allow this transition to occur. Strand β2 itself can feel this twist leading to a global “breathing” of the β‐sheet. ^15^N‐Δω_M‐M*_ values are large, ranging from 2.5 to 9 ppm for both the β2 strand and the hairpins (Figure 3D), and ^1^H_N_‐Δω values are also significant (≈1 ppm), suggesting large changes in backbone dihedral angles and/or hydrogen bonding patterns. Since the ^15^N off‐resonance *R*_1ρ_ experiment (Figure 3B) also allows the determination of the sign of Δω, comparisons of the chemical shifts of species M* with the corresponding random coil values can be made, as shown in Figure 4B. A high correlation between these two quantities is observed, with the chemical shifts of M* being ≈25 % (2 ppm) smaller than their random coil values, indicating that state M* is partially folded.

It is perhaps surprising that numerous residues spanning the entire CTD structure show μs dynamics, as shown in Figure 3, with the T193A mutation enhancing these motions for residues that may be more than 40 amino acids away in the linear sequence. The relaxation dispersion data for all residues can be well fit using a single exchange rate (Figure 3, and Figures S2–S4), an observation that further supports the idea of concerted motions across the entire β‐sheet structure. To obtain additional insights into how alteration of the backbone conformation in strand β2 may affect the structure of the CTD as a whole, we developed a pseudo‐energy potential term implemented in XPLOR‐NIH,^[21]^ termed twistPot. TwistPot can alter the backbone twist[^9b^, ^22^] (Figure S6A) of any 4‐residue window while ensuring agreement with the input NMR experimental data. Changing the twist angle of the central 4‐residue window of the β2 strand (residues 203–206) by a modest 20° (or 60°) triggered a global conformational change to the structure of the CTD, causing it to adopt a flatter β‐sheet configuration (Figures 4C and S6B, C). This is particularly picked‐up by residues in the hairpin regions as they feel the backbone re‐orientation of both connecting β‐strands. Hairpins in the CTD are especially interesting as they all comprise the same [ND]G[RKQ] consensus sequence, which has a strong preference for random coil in the ProteinDataBank (rcsb.org)^[23]^ (72 % of 1926 hits). However, when this sequence is found in a secondary structure element, it almost exclusively adopts a typical type I′ turn structure found in hairpins (19 % turn I′, 5 % helix, 3 % turn II,1 % turn I). The secondary chemical shifts of these residues in the hairpin conformation are particularly large, explaining the large Δω values seen in Figure 3D.

The T193A substitution appears to reduce the ability of the β2 strand to adopt a specific backbone configuration, at least on the μs timescale. As a consequence, these backbone fluctuations should be reflected in the ability of backbone amide groups to form hydrogen bonds. To test this hypothesis, we measured CTD hydrogen exchange rates using the DÉCOR experiment,^[24]^ which relies on the measurement of the relaxation rate (*k*_zz_) of H_z_N_z_ two‐spin order, which is dominated by exchange with solvent.^[24]^ As seen in Figure 4D, *k*_ZZ_ rates show large fluctuations expected for the outer strands β1 and β4, while the internal strands β2 and β3 are better protected from water exchange. However, residues T204 and T205 in the middle of strand β2 show increased *k*_zz_ rates compared to the other strand β2/β3 residues, especially in the context of the T193A mutation (Figures 4D and S6D). This observation suggests that enhanced backbone motions experienced by the T193A mutant lead to transient breaking of inter‐strand hydrogen bonds, consistent with the relatively large ^1^H_N_ Δδω values for residues in strand β2 (Figure 1A) and the ≈1 ppm values for ^1^H_N_‐Δω_M‐M*_ (Table S3).

In conclusion, chaperone oligomerization is a fundamental aspect of chaperones that are effective against client protein aggregation.^[1]^ The CTD of DNAJB6b and the Parkinson's disease‐related mutation, T193A,^[11]^ provide a unique opportunity to explore the link between chaperone oligomerization, substrate binding, and onset of disease. Here, to study DNAJB6 oligomerization without having to take into account additional equilibria related to inter‐domain interactions that take place on a similar timescale,^[9a]^ we have used constructs of the isolated CTD. Using an array of relaxation‐based NMR techniques, we were able to probe μs fluctuations which may correspond to backbone crankshaft motions^[20]^ which are enhanced in the T193A mutant. These motions lead to the formation of a partially‐folded state that is off‐pathway to DNAJB6 oligomerization and reduces the ability of the T193A mutant to form functional oligomers. Indeed, mutational studies described in ref. [10] showed that substituting residues 190–195 to alanine substantially reduces DNAJB6′s anti‐aggregation activity. Our structural analysis suggests that even a small change in the twist of a β‐strand can change the strength of inter‐strand hydrogen bonds, leading to global effects reflected in the flatness of the entire β‐sheet, which in turn controls its ability to self‐assemble. How the low complexity, disordered region that lies N‐terminal to the CTD affects CTD oligomerization still needs to be investigated. Overall, backbone corelated motions that may lead to slower dynamics observed in strand β1 may play an important role in modulating CTD self‐assembly, a phenomenon that has been proposed to play a role in other β‐sheet mediated interactions, such as amyloid formation.^[25]^ In conclusion, our study provides a mechanistic understanding of how DNAJB6 mutations may lead to disease by modulating functionally important chaperone oligomerization.

## Conflict of interest

The authors declare no conflict of interest.

## Supporting information

As a service to our authors and readers, this journal provides supporting information supplied by the authors. Such materials are peer reviewed and may be re‐organized for online delivery, but are not copy‐edited or typeset. Technical support issues arising from supporting information (other than missing files) should be addressed to the authors.

Supporting Information

Supporting Information

## Data Availability

The data that support the findings of this study are openly available on figshare (DOI: 10.6084/m9.figshare.17096675, reference number 26).
