# Supplementary material for: Microsecond Backbone Motions Modulate the Oligomerization of the DNAJB6 Chaperone
Source: Angew Chem Weinheim Bergstr Ger. 2022 Mar 19;134(20):e202116403. doi: 10.1002/ange.202116403 (PMC10947091; doi:10.1002/ange.202116403)
Supplement: Supplementary file 1 — Supporting Information [file ANGE-134-0-s001.pdf]

Angewandte Chemie
Eine Zeitschrift der Gesellschaft Deutscher Chemiker

Supporting Information

Microsecond Backbone Motions Modulate the Oligomerization of the DNAJB6 Chaperone

E. E. Cawood, G. M. Clore, T. K. Karamanos**

SUPPORTING INFORMATION

Microsecond Backbone Motions Modulate the Oligomerization of the DNAJB6 Chaperone

Emma E. Cawood,[a, c] G. Marius Clore*[b] and Theodoros K. Karamanos*[a]

[a]Astbury Centre for Structural Molecular Biology, School of Molecular and Cellular Biology University of Leeds, Mount Preston Street, Leeds, LS2 9JT, U.K.

[b]Laboratory of Chemical Physics, National Institute of Diabetes and Digestive and Kidney Diseases, National Institutes of Health, Bethesda, MD, 20892-0520, U.S.A.

[c]Current address: Crick-GSK Biomedical LinkLabs, GlaxoSmithKline, Gunnel Wood Road, Stevenage, SG1 2NY

*E-mail addresses: G.M.C., mariusc@mail.nih.gov; T.K.K., t.karamanos@leeds.ac.uk

SI Materials and Methods

Protein expression and purification. The WT CTD and T193A CTD gene constructs were expressed and purified as described previously.[1]

NMR spectroscopy and collection of nuclear Overhauser enhancement (NOE) data. Assignment of the T193A backbone resonances that are significantly altered in comparison to the WT CTD spectrum were obtained from analysis of 3D triple resonance through-bond correlation spectra (HNCACB, CBCACONH, and HBHACONH) recorded at 600 MHz on a sample of 200 μM U-[$^{15}N/^{13}C$]-labeled T193A CTD in 20 mM sodium phosphate, pH 6.3, 150 mM NaCl, 95% (v/v) H_2O/5% (v/v) D_2O. All NMR experiments were performed under the same conditions unless otherwise stated. The 3D NOE experiments (mixing time = 200 ms) used for structure determination were as follows: 3D HCH-NOE-HSQC (600 MHz) with a 28 ms constant time ^1H-^{13}C HSQC readout, non-uniformly sampled at a 20% level with ^1H t_1 and ^{13}C t_2 acquisition times of 25 and 28 ms, respectively; 3D HCC HMQC-NOE-HSQC (600 MHz) with a 28 ms constant time ^1H-^{13}C HSQC readout, non-uniformly sampled at a 20% level with ^{13}C t_1 and ^{13}C t_2 acquisition times of 10 and 28 ms, respectively. Spectra were processed with NMRPipe [2] and analysed using CCPNMR.[3] The program SMILE[4] was used to reconstruct the non-uniformly sampled spectra and double the acquisition times in the indirectly detected dimensions of the uniformly sampled spectra. Water suppression was achieved using magic angle gradients for coherence selection.[5]

^{15}N-CPMG relaxation dispersion measurements. ^{15}N CPMG relaxation dispersion experiments were recorded at 600 and 800 MHz using a pulse scheme with amide proton decoupling to measure the rates of in-phase ^{15}N coherences.[6] The constant time period for the CPMG sequence was set to 40 or 60 ms. ^1H_N constant wave (CW) decoupling was applied at a radiofrequency (RF) field strength of 11 kHz. The same experiment recorded with the relaxation period omitted served as a reference for the calculation of $R_{2,\mathrm{eff}}$ rates as a function of ν_{CPMG}, as described previously.[7] Uncertainties in $R_{2,\mathrm{eff}}$ values were obtained from duplicate measurements at two different ν_{CPMG} frequencies.

On-resonance ^{15}N-$R_{1\rho}$ measurements. ^{15}N-$R_{1\rho}$ and ^{15}N-R_1 measurements at 600, 750, and 800 MHz were recorded in an interleaved manner using heat-compensated ^1H-^{15}N HSQC-based pulse schemes[8] on [^{15}N - ^3C]-labeled WT and T193A CTD samples over a range of concentrations. ^{15}N-R_2 (in-phase) values in the rotating frame were determined from the relationship $R_2 = (R_{1\rho} - R_1\cos^2\theta)/\sin^2\theta$, where θ is the angle between the effective spin-lock field and the external magnetic (B_0) field.

Off-resonance ^{15}N-$R_{1\rho}$ measurements. Off-resonance ^{15}N-$R_{1\rho}$ experiments were performed using the sequence of Yuwen *et al.*[9] with a 2 kHz spin-lock RF field strength and recorded as a pseudo-4D dataset with spin-lock times of 10 and 80 ms for each offset (spanning from -4000 to +4000 Hz), with the carrier set at 121.5 ppm. The error in the $R_{1\rho}$-R_1 values was calculated using a Monte Carlo approach based on the estimated average spectral noise and was propagated to the $R_{2,\mathrm{eff}} - R_1$ values using the relationship $\sigma_{R2\text{-}R1} = \sigma_{R1\rho - R1}/\sin^2(\theta)$ where θ is the angle between the spin-locked ground state magnetization and the z axis.[9]

On-resonance ^{1}H$_N$-$R_{1\rho}$ measurements. On-resonance ^1H_N-$R_{1\rho}$ experiments were performed using a modified version of the pulse sequence of Skrynnikov *et al.*,[10] in which the sensitivity enhancement element was excluded. The ^1H carrier was placed at the centre of the amide region (8.5 ppm) before the magnetization was aligned with the effective field using adiabatic pulses (4 ms). Three spin lock times (5, 20, 50 ms) were recorded for each spin lock field strength (spanning from 1000 to 5000 Hz). Relaxation

rates and their errors were extracted by fitting single exponentials to the resulting data for each spin lock field strength.

Measurement of $^{3}J_{HN\text{-}H\alpha}$ couplings. $^{3}J_{HN\text{-}H\alpha}$ couplings were measured using the ARTSY-based method as described previously.[11] The value of $^{3}J_{HN\text{-}H\alpha}$ is calculated as $c[\cos^{-1}(I_A/I_B)\pi t_d)$ where I_A and I_B are the intensities in reference and attenuated (where the coupling is active for t_d = 30 ms) spectra, respectively, and c is a scale factor that depends upon R_1 and t_d, and was estimated to have a value of 1.04.

Measurement of k_{ZZ} rates. k_{ZZ} rates were measured using the DÉCOR experiment of Skrynnikov and Ernst[12] at 298 K. Briefly, after generation of H_zN_z two-spin order using a standard INEPT transfer, a delay is inserted (τ_{DECOR}) during which exchange of the amide proton with solvent serves to break up the correlation with ^{15}N, resulting in signal loss. T_1 relaxation also takes place during τ_{DECOR} but $R_{1,HzNz}$ rates are reasonably assumed to be the same for the WT and T193A CTDs, and therefore the differences in k_{ZZ} rates shown in Figure 4D reflect differences in water exchange rates between the two proteins. As a control, we also performed hydrogen exchange experiments in which the water resonance was inverted selectively that also revealed elevated hydrogen exchange rates for residues in the β2 strand.

Structure calculation and refinement. Simulated annealing calculations were carried out in XPLOR-NIH[13] to refine the structure of the T193A CTD mutant, starting from the refined WT CTD structure (PDB ID: 7JSQ). A total of 222 unambiguously assigned NOEs were used, with 8 of these involving protons of A193. NOEs were classified as strong (<2.5 Å), medium (<3.5 Å) or weak (<4.5 Å), with an extra 1 Å added to the restraint if one of the two protons involved originated from a methyl group. The target function included terms for the experimental NOE-derived interproton distance restraints, $^{3}J_{HN\text{-}H\alpha}$ couplings, and Talos-derived[14] backbone torsion angle restraints. Two statistical potentials of mean force were also included in the target function: torsionDB,[15] to ensure stereochemically reasonable backbone and side-chain torsion angles, and HBPot,[16] to optimize hydrogen bonding interactions. Control calculations showed that HBPot does not bias the resulting structures but does improve structure quality. All simulated annealing calculations were carried out in torsion angle space. The first stage in the structure calculation consisted of 5000 steps of energy minimization, followed by simulated annealing torsion angle dynamics with all the potential terms active. During the high temperature phase (T = 3000 K), the NOE terms were underweighted to allow sampling of a large region of conformational space, and then geometrically increased during the cooling phase (3000 to 25 K).

TwistPot. To calculate the backbone twist from atomic coordinates, we use four consecutive Cα atoms and define the midpoints between Cα(i)–Cα(i+1), Cα(i+1)–Cα(i+2), and Cα(i+2)–Cα(i+3) as M1, M2, and M3, respectively. P is the midpoint between M1–M2, and Q is the midpoint between M2–M3. The calculated twist angle (D_{calc}) is then defined as the dihedral angle between Cα(i+1), P, Q, and Cα(i+2) (see Figure S6A), making sure that the twist angle of the trans state is 0°. The pseudo-potential that was incorporated into XPLOR-NIH[13] was therefore defined as:

$$E_{twist} = k_{twist} \sum_{m}^{N} diff \tag{S1}$$

where

$$diff = \begin{cases} D_{\text{calc}} - D_{\text{target}} \text{ if } D_{\text{calc}} > D_{\text{target}} \\ D_{\text{target}} - D_{\text{calc}} \text{ if } D_{\text{calc}} < D_{\text{target}} \end{cases} \tag{S2}$$

D_{target} is the desired twist angle in degrees, N the number of restraints, and k_{twist} a force constant (set to a high value of 1000 kcal^{-1}mol^{-1}, since $N = 1$ here). D_{target} of the central β2 strand window was set to a series of values ranging from 4° to 64° (the value observed in the refined T193A CTD structure is ~20°). TwistPot was used in combination with all the experimental (NOE, $^3J_{HN\text{-}H\alpha}$, φ, ψ torsion angles), and geometrical and statistical (torsionDB, HBpot) terms, in an effort to introduce backbone twists and investigate the conformational changes needed to accommodate them, while preserving the overall architecture of the β-sheet. TwistPot and associated TwistPotTools are freely available at karamanoslab.com/resources.

Global fitting of relaxation-based data to a 4-state kinetic model. Concentration-independent ^{15}N-CPMG, off-/on-resonance ^{15}N-$R_{1\rho}$, and on-resonance ^{1}H$_N$-R$_{1\rho}$ relaxation dispersion data were globally fit to 4-state model shown in Figure 4A and Scheme S1. The relevant equations for the linear 4-state kinetic model involving interconversion between monomer (M), dimer (D), monomer excited state (M*), and oligomer-bound dimer (DO) species are described in detail in ref. (1). The only differences relate to the incorporation of the monomeric state M* instead of the oligomer-bound monomer state MO in ref. (1). Removing MO is justified since the data in ref. (1) can be fitted almost equally well to the on-pathway 3-state model $M \leftrightarrow D \leftrightarrow DO$.

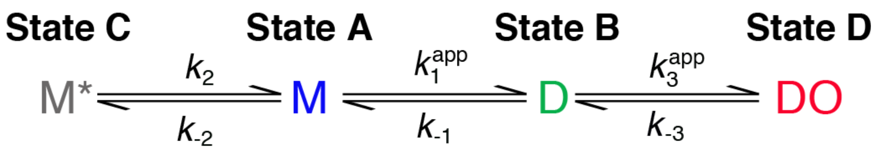

Scheme S1

M, D, M*, and DO are equated with states **A**, **B**, **C**, and **D** respectively. State **A** is the major species, while states **B**, **C**, and **D** are sparsely-populated. State **B**, the dimer, is assumed to have a transverse relaxation rate twice that of state **A**, the monomer: $R_2^B = 2R_2^A$. State **C**, the excited-state monomer and state **D**, the oligomer-bound dimer have transverse relaxation rates R_2^C and R_2^D, respectively with $R_2^C = R_2^A$. The longitudinal relaxation rates for states **B** (R_1^B), **C** (R_1^C), and **D** (R_1^D) were assumed to be the same as those of the experimentally determined values for state **A**. k_2 is a first order rate constant, k_1^{app} and k_3^{app} are apparent pseudo-first order association rate constants, and k_{-1}, k_{-2} and k_{-3} are first order dissociation rate constants. All states were assumed to be present at the start of the experiment $M(0) = (p_{A,}, p_B, p_C, p_D)$ where p_N is the equilibrium population of state N. The population of the main state, **A**, is:

$$p_A = \frac{1}{1 + \frac{k_1^{app}}{k_{-1}} + \frac{k_2}{k_{-2}} + \frac{k_1^{app}k_3^{app}}{k_{-1}k_{-3}}} \qquad \text{(S3)}$$

and the remaining populations are given by:

$$p_B = \frac{k_1^{app}}{k_{-1}} p_A\,,\; p_C = \frac{k_2}{k_{-2}} p_A\,,\; p_D = \frac{k_3^{app}}{k_{-3}} p_B \qquad \text{(S4)}$$

Parameters k_{-1}, k_{-3}, k_1^{app}, k_3^{app}, R_2^{B}, R_2^{D}, $\Delta\bar{\omega}_{\mathrm{B}}$, $\Delta\bar{\omega}_{\mathrm{D}}$ (the difference in chemical shifts between states **A** and **B**, and **B** and **D**, respectively) were fixed to their optimised values as described previously[1], leaving k_2, k_{-2}, R_2^{A}, and $\Delta\bar{\omega}_{\mathrm{C}}$ as fitting parameters.

Since the **A** ↔ **C** interconversion is fast on the chemical shift timescale, $\Delta\bar{\omega}_{\mathrm{C}}$ (the difference in chemical shifts between states **A** and **C)** and p_C are correlated and cannot be deconvoluted using $R_{1\rho}$ data alone. The latter, however, provide a very good estimate of R_2^{A}. To overcome this issue, we incorporated ^{15}N-CPMG relaxation dispersion data, which, for fast exchange rates (>5000 s^{-1}), decrease monotonically as a function of the pulse frequency (Figure S5B). Numerical simulations and fits to well-digitised $R_{1\rho}$ and CPMG relaxation dispersion data generated using a 3% population for the excited state (p_E), with $\Delta\bar{\omega}_E$ = 6 ppm and R_2^{A} = 7 s^{-1}, are shown in Figure S5. Off-resonance $R_{1\rho}$ data at various spinlock field strengths can be fit within error, even if p_E is fixed at a high value of 25% (Figure S5A). The fit to the CPMG relaxation dispersion curves is also reasonable, although the optimized value of $R_{2,\mathrm{CPMG}}^{\mathrm{A}}$ is unrealistically low (~1 s^{-1}) in comparison with $R_{2,\mathrm{R}1\rho}^{\mathrm{A}}$, which is much closer to the correct value of 7 s^{-1} (Figure S5A). Restraining $R_{2,\mathrm{CPMG}}^{\mathrm{A}} \sim R_{2,\mathrm{R}1\rho}^{\mathrm{A}}$ results in a much poorer fit to the CPMG relaxation dispersion data and thus provides a useful upper limit on p_E. Thus, when fitting actual experimental data, $R_{2,\mathrm{CPMG}}^{\mathrm{A}}$ was set to be within ± 2 s^{-1} of $R_{2,\mathrm{R}1\rho}^{\mathrm{A}}$. In the case of the T193A mutant, very low values for p_C were excluded based on unrealistically high values for the chemical shifts of amide protons, which exceed their chemical shift dispersion in the Biological Magnetic Resonance Bank (BMRB). Using this strategy, values of p_C and $\Delta\bar{\omega}_{\mathrm{C}}$ are relatively well-defined for both WT CTD and the T193A mutant (Figure S5C). The ^{15}N-CPMG, ^{15}N-$R_{1\rho}$, and ^{1}H$_{\mathrm{N}}$-$R_{1\rho}$ relaxation dispersion data were fit simultaneously by minimizing the sum of square differences between observed (*obs*) and calculated (*calc*) values, using an in-house Python script that employs the lmfit module.[17]

Data avaliability. The 10 lowested energy structural models of T193A CTD were deposited in the ProteinDataBank, PDB ID 7QBY. T193A CTD backbone chemical shifts were deposised in the BMRB, ID 34686. NMR relaxation data and processing scripts were deposited in digital format on figshare (doi: 10.6084/m9.figshare.17096675).

Supplementary Figures

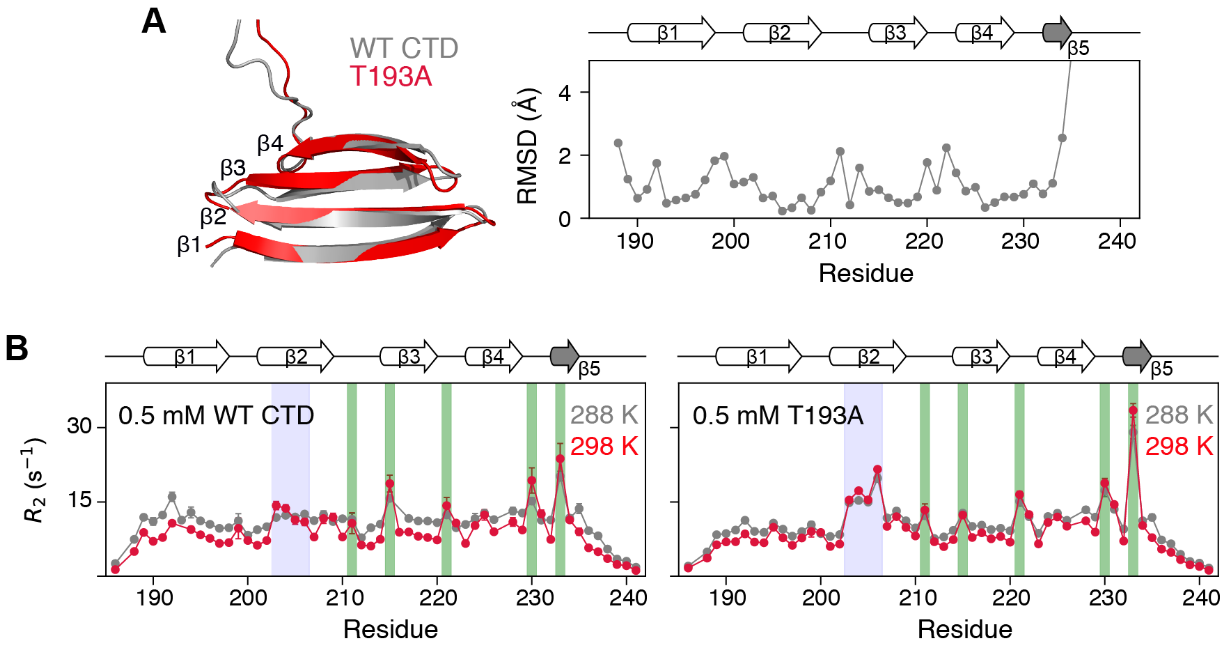

Figure S1. (A) Comparison of WT and T193A CTD structures. Left, overlay of the lowest energy WT (grey) and T193A (red) structures. Right, Cα RMSD between the two structures. (B) ^{15}N-R_2 profiles for 0.5 mM WT CTD and 0.5 mM T193A CTD, at 288 K (grey) and 298 K (red).

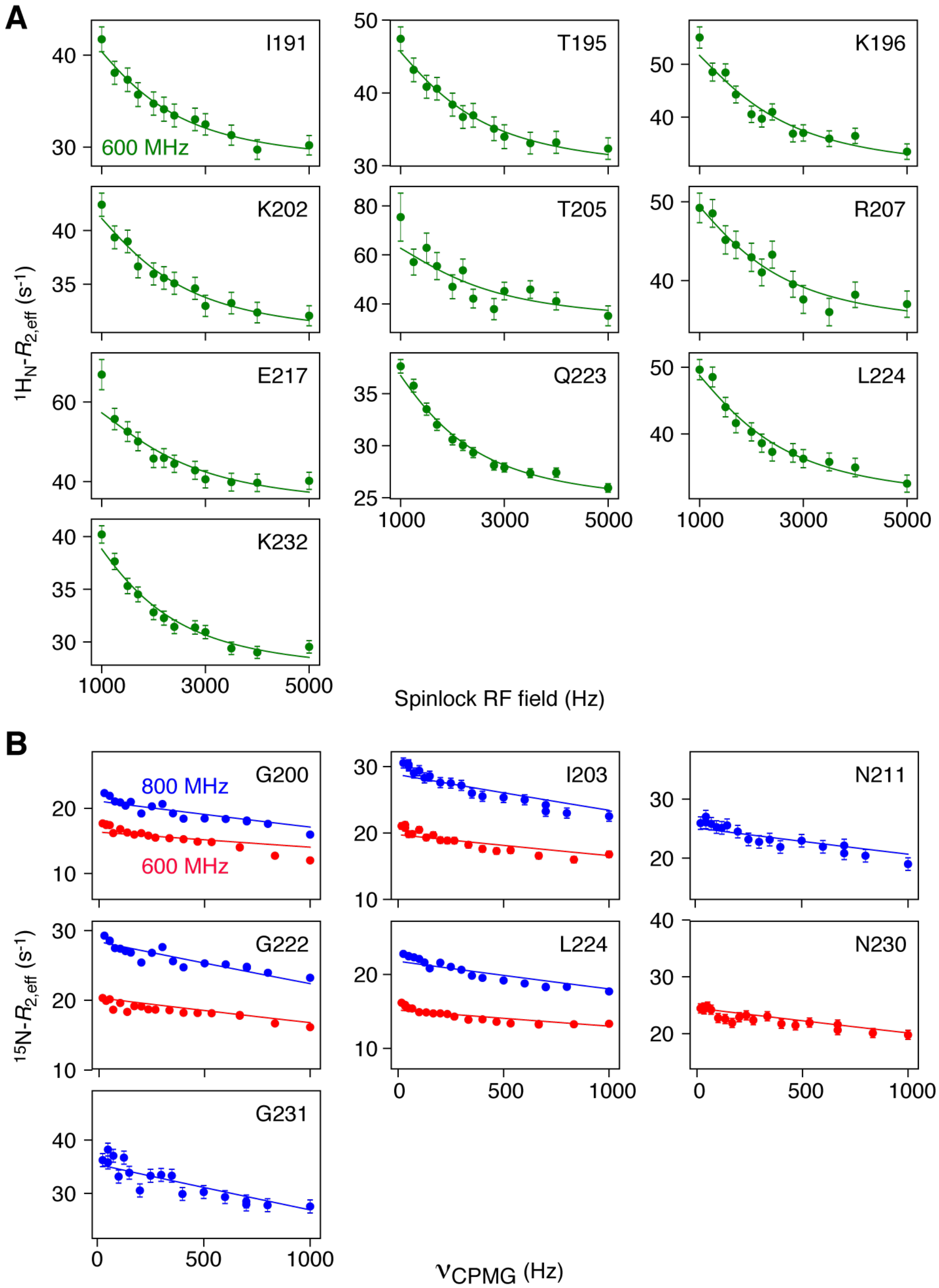

Figure S2. Dynamics of the T193A CTD mutant probed by on-resonance $^{1}H_{N}$-$R_{1\rho}$ and ^{15}N-CPMG relaxation dispersion. (A) On-resonance $^{1}H_{N}$-$R_{1\rho}$ and (B) ^{15}N-$R_{2,eff}$ CPMG profiles as a function of spinlock and CPMG field strengths, respectively. The experimental data are displayed as circles. The continuous lines represent global fits to the 4-state model shown in Scheme S1 (and Figure 4A). The data were recorded at 288 K and 600 or 800 MHz on a 0.5 mM sample of U-[^{15}N/^{13}C/^{2}H]-labeled T193A mutant in 20 mM sodium phosphate, pH 6.7, 150 mM NaCl, 95% (v/v) H_2O/5% (v/v) D_2O.

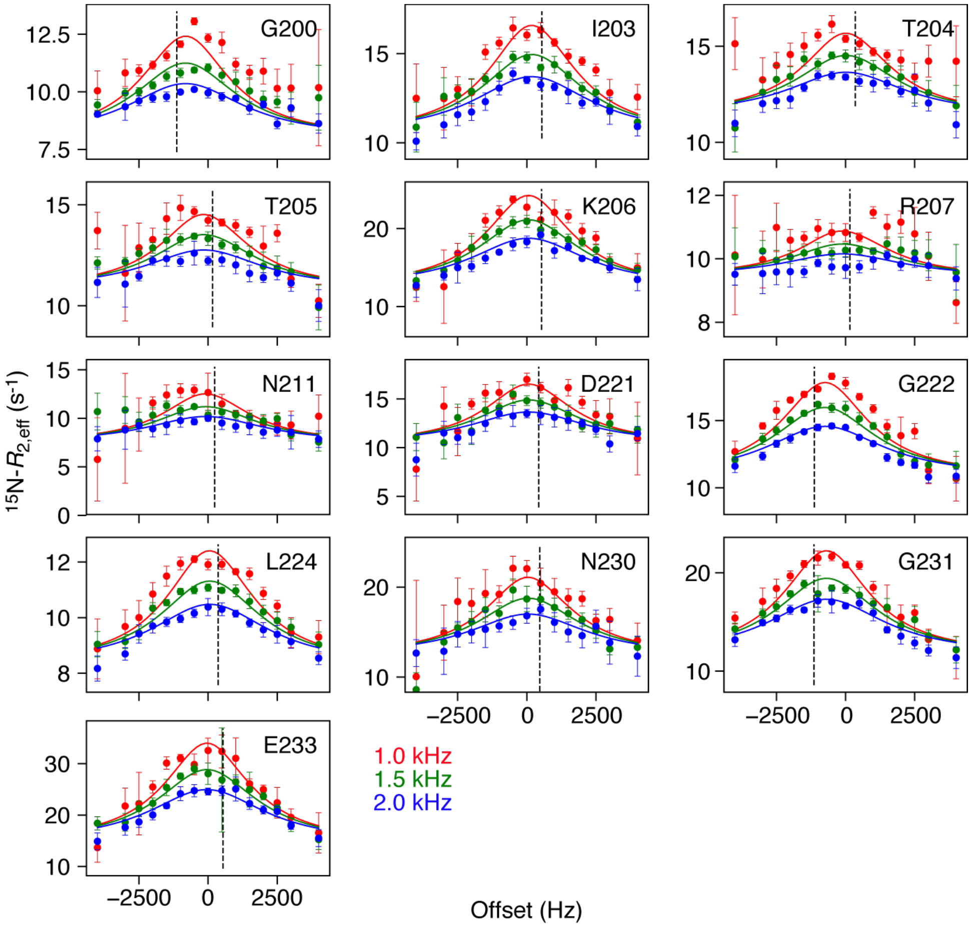

Figure S3. Dynamics of the T193A CTD mutant probed by off-resonance ^{15}N-$R_{1\rho}$ dispersion. ^{15}N off-resonance $R_{1\rho}$ profiles are shown as a function of offset from the carrier frequency at various spinlock field strengths. The offset from the carrier frequency of the major monomeric species is shown as a black dashed line. The experimental data are displayed by circles. The continuous lines represent global fits to the 4-state model shown in Scheme S1 (and Figure 4A). The data were recorded at 288 K and 600 MHz on a 0.5 mM sample of U-[^{15}N/^{13}C/^{2}H]-labeled T193A mutant in 20 mM sodium phosphate, pH 6.7, 150 mM NaCl, 95% (v/v) H_2O/5% (v/v) D_2O.

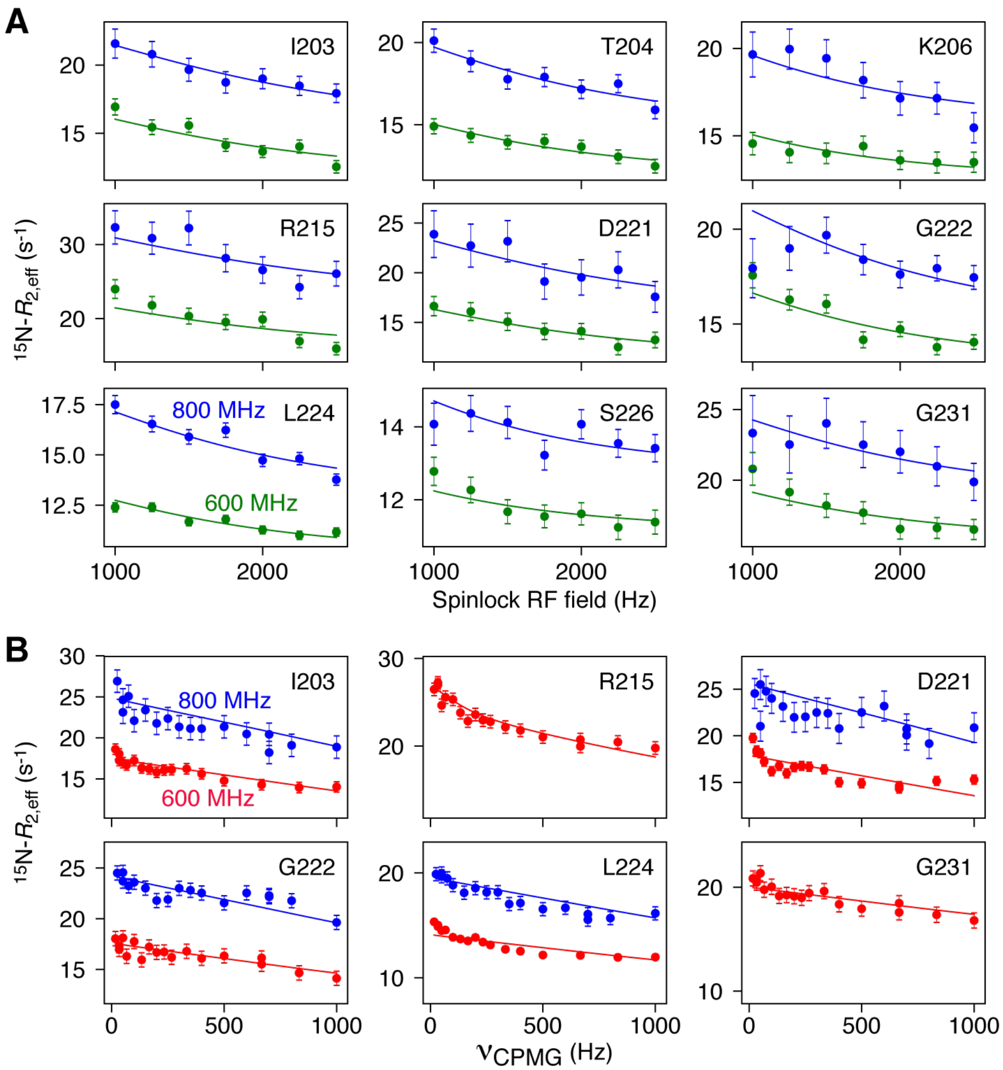

Figure S4. Probing μs dynamics of WT CTD. (A) On-resonance ^{15}N-$R_{1\rho}$ and (B) ^{15}N-CPMG relaxation dispersion profiles, recorded at 288 K. Data were collected at 800 (blue) and 600 (red) MHz on a 0.5 mM sample of U-[^{15}N/^{13}C/^{2}H]-labeled WT CTD in 20 mM sodium phosphate, pH 6.7, 150 mM NaCl, 95% (v/v) H_2O/5% v/v D_2O. Continuous lines represent fits to the 4-state model of Scheme 1 (and Figure 4A).

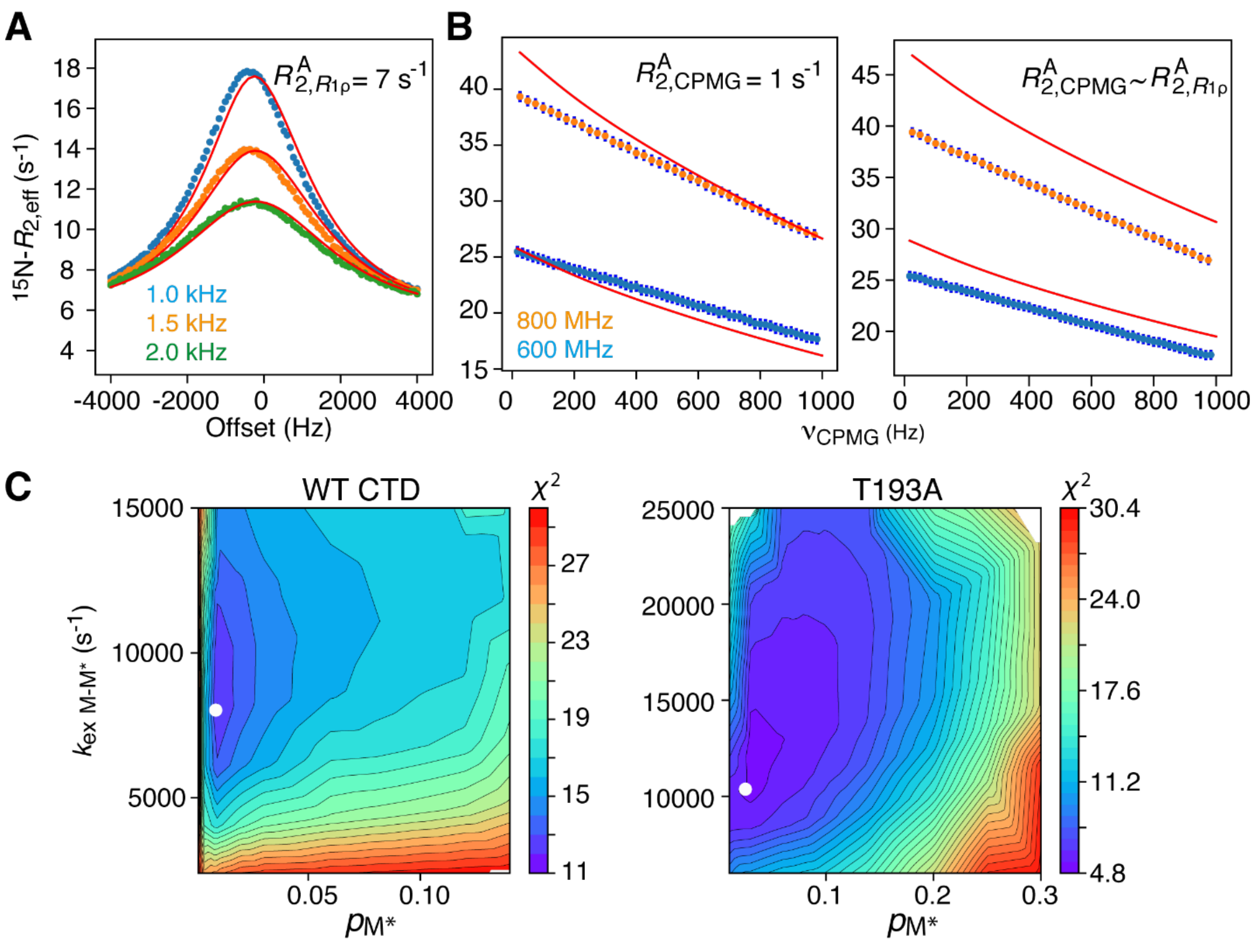

Figure S5. Deconvoluting p_E and $\Delta\bar{\omega}_E$. Dots represent (A) simulated off-resonance ^{15}N-$R_{1\rho}$ and (B) ^{15}N-CPMG relaxation dispersion profiles. These profiles were simulated using p_E = 3%, $\Delta\bar{\omega}_E$ = 6 ppm, and R_2^{A} = 7 s^{-1}. Continuous lines represent fits to the simulated data with p_E fixed to 25%. In the left panel in (B), two different parameters for the R_2^{A} in each experiment were fitted ($R_{2,\mathrm{CPMG}}^{\mathrm{A}}$, $R_{2,\mathrm{R1}\rho}^{\mathrm{A}}$) with their values optimized to 1 and 7 s^{-1}, respectively. In the right panel, an additional restraint is imposed for $R_{2,\mathrm{CPMG}}^{\mathrm{A}} \sim R_{2,\mathrm{R1}\rho}^{\mathrm{A}}$. (C) Heat maps of the fitted k_{ex} values for the M ↔ M* transition versus p_{M*}, using the data of Figures S2-S4 and the model of Figure 4A, colored by the reduced χ^2 for (left) WT CTD and (right) T193A CTD. The optimised values of k_{ex} and p_{M*} are shown as white dots.

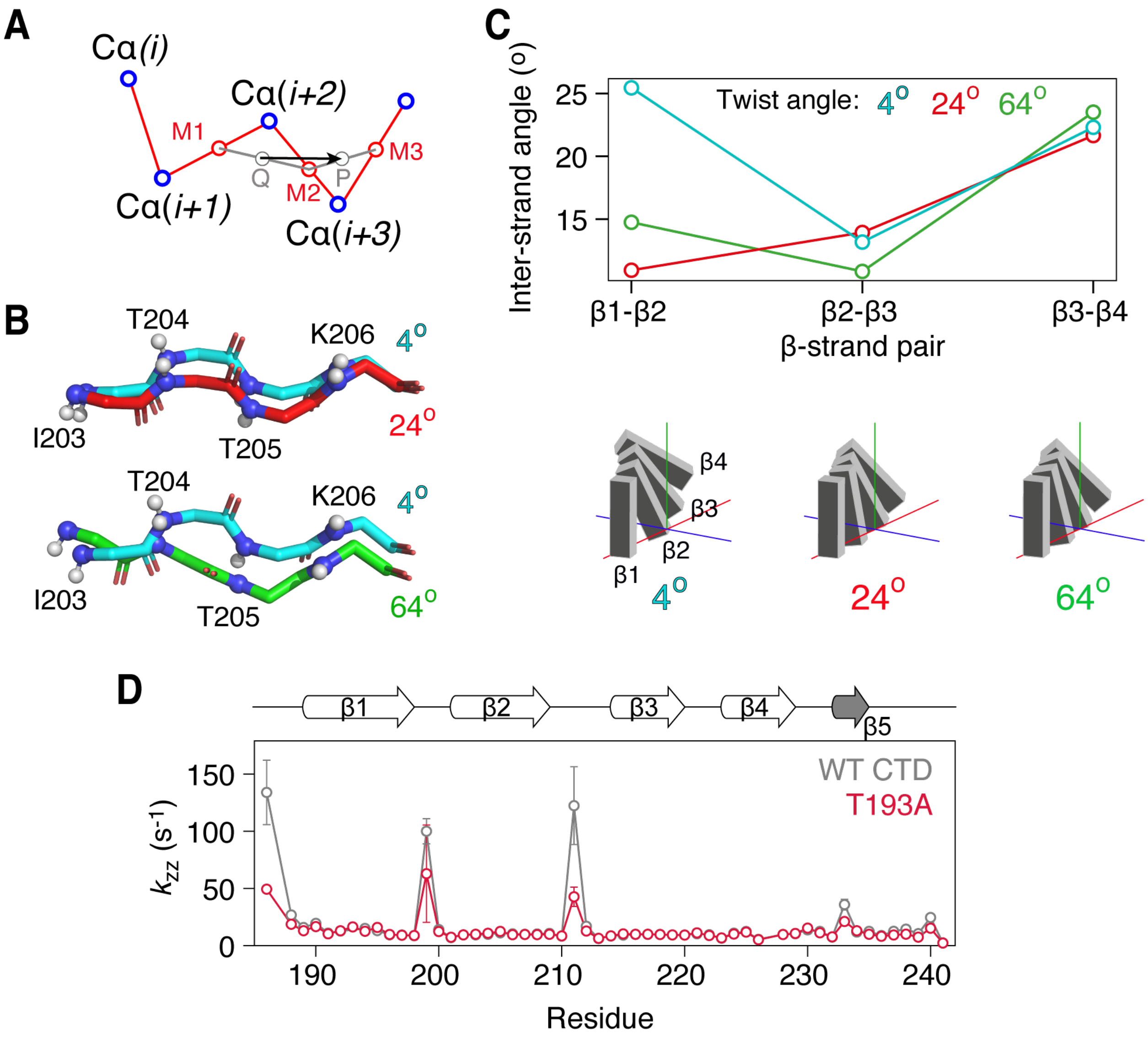

Figure S6. Structural analysis of the CTD. (A) Definition of the backbone twist angle. For four consecutive Cα atoms, we define the midpoints between Cα(*i*)–Cα(*i*+1), Cα(*i*+1)–Cα(*i*+2), and Cα(*i*+2)–Cα(*i*+3) as M1, M2, and M3, respectively. P is the midpoint between M1–M2, and Q is the midpoint between M2–M3. The twist angle is then defined as the dihedral angle between Cα(*i*+1), P, Q, and Cα(*i*+2). The twist angle of the trans state is 0°. (B) Stick representation of the backbone atoms of the central window of the β2 strand when the corresponding twist angle is set to 4° (cyan, top and bottom), 24° (red, top) or 64° (green, bottom) using twistPot. (C) Angles between consecutive β-strand pairs (top) and a schematic of the overall CTD configuration (bottom) when the twist angle of residues 203-206 in strand β2 is set to 4° (cyan), 24° (red), or 64° (green). In the schematic, each β-strand is shown as a grey box rotated by the corresponding angle shown in the diagram. (D) Zoomed-out view of the hydrogen exchange data shown in Figure 4D. k_{ZZ} rates were obtained using the DÉCOR experiment[10] at 298 K and 600 MHz as a function of residue number for WT (grey) and T193A (red) CTD.

**Table S1.** Structural statistics (PDB 7QBY)	
R.m.s deviations from experimental distance restraints (Å)	
Total (222)	0.181 ± 0.02
R.m.s deviations from expt. 3-bond $J_{HN\text{-}H\alpha}$ restraints (degrees) (48)	1.182 ± 0.09
R.m.s deviations from expt. dihedral restraints (degrees) (97)	6.77 ± 2.28
Deviations from idealized covalent geometry	
Bonds (Å) (885)	0.001 ± 0.000
Angles (°) (1609)	0.329 ± 0.000
Impropers (°) (420)	0.948 ± 0.103
Measures of structure quality[1]	
Residues in most favorable Ramachandran regions	96 ± 2 %
Ramachandran outliers	0 ± 0 %
Number of bad contacts per 1000 atoms (H included)	17
Residues with favorable side-chain rotamers	100 ± 1 %
Structure precision[2]	
Backbone RMSD (Cα) to mean coordinate positions	1.15± 0.08

[1]From the PDB validation server
[2]Reported for residues 137-180

Table S2. Experimental data used in global fits to relaxation-based NMR data

**Experiment**	**Magnetic field (MHz)**		**Residue number**	
**Protein**	**WT CTD**	**T193A CTD**	**WT CTD**	**T193A CTD**
**^{15}N-CPMG relaxation dispersion**	600, 800	600, 800	203, 215[a], 221, 222, 224, 231	200, 203, 211[a], 222, 224, 230
**^{15}N-on resonance $R_{1\rho}$**	600, 800	-	203, 204, 206, 215, 221, 222, 224, 226, 231	-
**$^{1}H_{N}$-on resonance $R_{1\rho}$**	-	600	-	191, 195, 196, 202, 205, 206, 217, 223, 224, 231
**^{15}N-off-resonance $R_{1\rho}$**	-	600	-	200, 203, 204, 205, 206, 207, 211, 221, 222, 223, 230, 231, 233

[a]CPMG data only at 600 MHz for these residues

Table S3. Fitted chemical shifts[a]

Residue/Atom	$\Delta\omega_{M-M*}$	
	**WT CTD**	**T193A CTD**
200 N	-	4.3 ± 0.11
203 N	8.5 ± 0.41	-5.1 ± 0.14
204 N	6.8 ± 0.28	-4.27 ± 0.13
205 N	-	-3.97 ± 0.12
206 N	5.21 ± 0.32	-6.98 ± 0.13
207 N	-	-2.54 ± 0 .01
211 N	-	-4.59 ± 0.16
221 N	9.0 ± 1.0	-5.07 ± 0.16
222 N	7.08 ±0.22	5.40 ± 0.15
223 N	6.31 ± 0.23	-4.16 ± 0.11
226 N	3.55 ± 0.15	-
230 N	-	-6.05 ± 0.20
231 N	6.8 ± 0.31	6.63 ± 0.17
233 N	-	-8.9 ± 0.23
191 H_N	-	0.72 ± 0.03
195 H_N	-	0.86 ± 0.04
196 H_N	-	1.04 ± 0.04
202 H_N	-	0.71 ± 0.03
205 H_N	-	1.29 ± 0.11
207 H_N	-	0.84 ± 0.04
217 H_N	-	1.15 ± 0.06
223 H_N	-	0.71 ± 0.02
224 H_N	-	0.94 ± 0.02
232 H_N	-	0.69 ± 0.02

[a]For the WT CTD and the H_N atoms of the T193A CTD mutant, the sign of $\Delta\omega$ is not determined

Supplementary References

[1] T. K. Karamanos, V. Tugarinov, G. M. Clore, *Proc. Natl. Acad. Sci. USA* **2020**, 202020306.
[2] F. Delaglio, S. Grzesiek, G. W. Vuister, G. Zhu, J. Pfeifer, A. Bax, *J. Biomol. NMR* **1995**, *6*, 277-293.
[3] W. F. Vranken, W. Boucher, T. J. Stevens, R. H. Fogh, A. Pajon, M. Llinas, E. L. Ulrich, J. L. Markley, J. Ionides, E. D. Laue, *Proteins* **2005**, *59*, 687-696.
[4] J. Ying, F. Delaglio, D. A. Torchia, A. Bax, *J. Biomol. NMR* **2016**, *68*, 101-118.
[5] D. L. Mattiello, Warren, L. Mueller, B. T. Farmer, *J. Am. Chem. Soc.* **1996**, *118*, 3253-3261.
[6] D. F. Hansen, P. Vallurupalli, L. E. Kay, *J. Phys. Chem. B* **2008**, *112*, 5898-5904.
[7] T. K. Karamanos, V. Tugarinov, G. M. Clore, *Proc. Natl. Acad. Sci. U. S. A.* **2019**, *116*, 21529-21538.
[8] N.-A. Lakomek, J. Ying, A. Bax, *J. Biomol. NMR* **2012**, *53*, 209-221.
[9] T. Yuwen, J. P. Brady, L. E. Kay, *J. Am. Chem. Soc.* **2018**, *140*, 2115-2126.
[10] C. Eichmüller, N. R. Skrynnikov, *J. Biomol. NMR* **2005**, *32*, 281-293.
[11] J. Roche, J. Ying, Y. Shen, D. A. Torchia, A. Bax, *J. Magn. Reson.* **2016**, *268*, 73-81.
[12] N. R. Skrynnikov, R. R. Ernst, *J. Magn. Reson.* **1999**, *137*, 276-280.
[13] C. D. Schwieters, G. A. Bermejo, G. M. Clore, *Protein Sci.* **2018**, *27*, 26-40.
[14] Y. Shen, F. Delaglio, G. Cornilescu, A. Bax, *J. Biomol. NMR* **2009**, *44*, 213-223.
[15] G. A. Bermejo, G. M. Clore, C. D. Schwieters, *Protein Sci.* **2012**, *21*, 1824-1836.
[16] C. D. Schwieters, G. A. Bermejo, G. M. Clore, *Protein Sci.* **2019**, *29*, 100-110.
[17] M. Newville, T. Stensitzki, D. B. Allen, A. Ingargiola, *zenodo.org*.
